# Electrochemical Degradation of Methylene Blue by a Flexible Graphite Electrode: Techno-Economic Evaluation

**DOI:** 10.1021/acsomega.2c04304

**Published:** 2022-09-02

**Authors:** Aysegul Yagmur Goren, Yaşar Kemal Recepoğlu, Özge Edebali̇, Cagri Sahin, Mesut Genisoglu, Hatice Eser Okten

**Affiliations:** † Department of Environmental Engineering, Izmir Institute of Technology, İzmir 35430, Turkey; ‡ Department of Chemical Engineering, Izmir Institute of Technology, İ zmir 35430, Turkey; § Environmental Development Application and Research Centre, İzmir Institute of Technology, İzmir 35430, Turkey

## Abstract

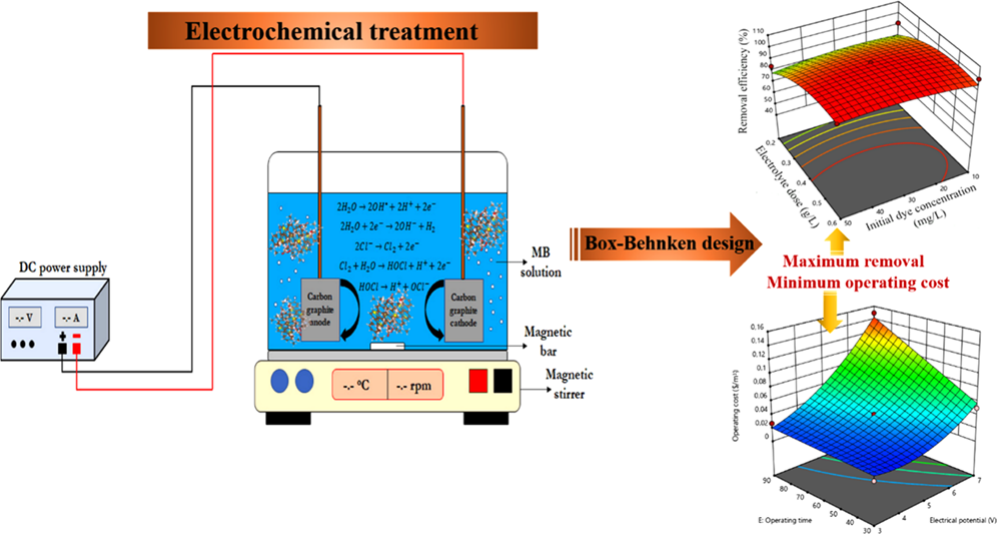

In this study, electrochemical removal of methylene blue (MB) from water using commercially available and low-cost flexible graphite was investigated. The operating conditions such as initial dye concentration, initial solution pH, electrolyte dose, electrical potential, and operating time were investigated. The Box-Behnken experimental design (BBD) was used to optimize the system’s performance with the minimum number of tests possible, as well as to examine the independent variables’ impact on the removal efficiency, energy consumption, operating cost, and effluent MB concentration. The electrical potential and electrolyte dosage both improved the MB removal efficiency, since increased electrical potential facilitated production of oxidizing agents and increase in electrolyte dosage translated into an increase in electrical current transfer. As expected, MB removal efficiency increased with longer operational periods. The combined effects of operating time–electrical potential and electrical potential–electrolyte concentration improved the MB removal efficiency. The maximum removal efficiency (99.9%) and lowest operating cost (0.012 $/m^3^) were obtained for initial pH 4, initial MB concentration 26.5 mg/L, electrolyte concentration 0.6 g/L, electrical potential 3 V, and operating time 30 min. The reaction kinetics was maximum for pH 5, and as the pH increased the reaction rates decreased. Consequent techno-economic assessment showed that electrochemical removal of MB using low-cost and versatile flexible graphite had a competitive advantage.

## 1 Introduction

The textile industry, as one of the most water-intensive industries, has exacerbated the water quality problem by utilizing a variety of synthetic dyes and discharging large amounts of highly colored wastewater, in addition to other minor issues such as solid waste management.^1−3^ Direct discharge of highly colored textile wastewater is an aesthetically unpleasant situation, but it also negatively impacts aquatic life by obstructing light penetration for photosynthetic function in plants.^4^ On the other hand, a large number of azo dyes and their breakdown products, such as component metals and chlorine, are toxic to or mutagenic for marine life.^5,6^ Methylene blue (MB) is one of the most frequently found organic dyes in textile wastewater.^7−9^ Several adverse effects have been reported at various doses based on exposure concentration, including skin desquamation and hemolytic anemia at 2–4 mg/kg, hemolysis, fever, chest pain, nausea, and vomiting at 7 mg/kg, hypotension at 20 mg/kg, and bluish discoloration at 80 mg/kg.^10^ As environmental regulations have become strict considering those side effects, MB in effluents must be treated prior to discharge.

There are widely used methods for the separation of MB from the environment, including biological processes,^11−13^ adsorption,^14−18^ membranes,^19−21^ ion exchange,^22^ coagulation processes,^23−26^ hybrid processes,^27−29^ and enhanced photocatalytic processes.^30−33^ However, these methods have drawbacks such as the high capital and operating costs required for membrane processes, high chemical consumption during coagulation/flocculation for pH adjustment and adsorption/ion exchange for sorbent regeneration, pollution transfer from one phase to another during adsorption/ion exchange, high sludge production during coagulation, the requirement for an enabling environment for biological processes, and the low removal efficiency of MB.^34,35^ As a result, enhancements to existing technologies are required to overcome these disadvantages. So far, electrochemical anodic oxidation processes (EAOPs) have received great interest for the treatment of aqueous media containing organic pollutants as they have advantages over their counterparts in terms of being eco-friendly, needing simple equipment and operation, no sludge formation, and relatively higher removal efficiencies.^36−39^

Recent studies have demonstrated that electro-oxidation is an efficient method for degrading organic pollutants when high-oxygen-overvoltage anodes such as boron-doped diamond (BDD),^40^ SnO_2_,^41^ and PbO_2_^42^ are used. In another study, although direct (using BDD) and indirect (by the active chlorine electrogenerated on the TiRuO_2_ oxide anode) electrochemical oxidation (EO) experiments resulted in complete oxidation of MB for 20 mA/cm^2^ current density, faster decolorization and mineralization of the solution was observed by indirect electrolysis, due to the high bleaching properties of active chlorine.^43^ Asghar et al. studied an EO technique consisting of electrodes made from stainless steel and graphite for mineralizing the MB dye dissolved in water.^44^ The results revealed a maximum dye removal of up to 80% with 40 min operation time and current density of 0.06 A/cm^2^. Alaoui et al. used Pt/MnO_2_ electrode as the anode and obtained higher than 90% MB removal at pH 8 with a current density of 7 mA/cm^2^ in the presence of less than 0.1 mol/L of sodium sulfate.^45^ To investigate the effects of chloride ions on the decolorization and mineralization of MB, a pre-pilot-scale electrochemical cell reactor comprising Ti/Pt and Ti/IrO_2_-Ta_2_O_5_ anodic materials was used in the presence or absence of chloride ions. Under the optimal treatment conditions of 40 mA/cm^2^ in 0.05 M of Na_2_SO_4_ at pH 6.0, solutions of 100 mg/L of MB were completely decolored, resulting in an 86.0% reduction in COD after 360 min of electrolysis at a cost of approximately 13.4 $/m3.^46^ Although the anode materials mentioned above are abundant in the literature, they are prohibitively expensive for industrial applications due to their inability to function as stable electrodes in large-scale EO processes. Additionally, several studies were conducted on the modification of those materials for use in laboratory-scale electrochemical treatment, but they were not commercially available. Therefore, low-cost and dimensionally stable anodes are required for sustainable large-scale applications.^47^ Recently, carbon-based materials have been employed as electrode materials in biochemical degradation processes as they are superconductive, environmentally benign, and nontoxic.^48^ To our best knowledge, there is limited study covering the electrochemical removal of MB from water using commercially available flexible graphite, which is a low-cost, highly conductive, and stable electrode material.

This is the most comprehensive study to date on the electrochemical removal of MB from water using flexible graphite electrodes. The effects of operating conditions such as initial concentration of MB (*C*_*i*,MB_), initial pH, electrolyte dose (*C*_*i*,NaCl_), electrical potential (EP), and operating time (*t*) were investigated to determine the optimum conditions. The Box-Behnken experimental design (BBD) was used to optimize the performance of the system with the minimum number of experiments, and to assess the influence of the variables on the removal efficiency (Re), energy consumption (ENC), operating cost (OC), and effluent MB concentration. Additionally, process outputs can be predicted without conducting experiments using BBD’s mathematical model, which offers a promising convenience for their application in real textile wastewater containing MB. A detailed investigation of the operational parameters driving electrochemical oxidation of an organic pollutant that is produced in high quantities and possible validation of a low-cost electrode material are the novel aspects of the present study.

### 1.1 Methylene Blue Removal Mechanism with Electrochemical Oxidation

**Figure 1:**
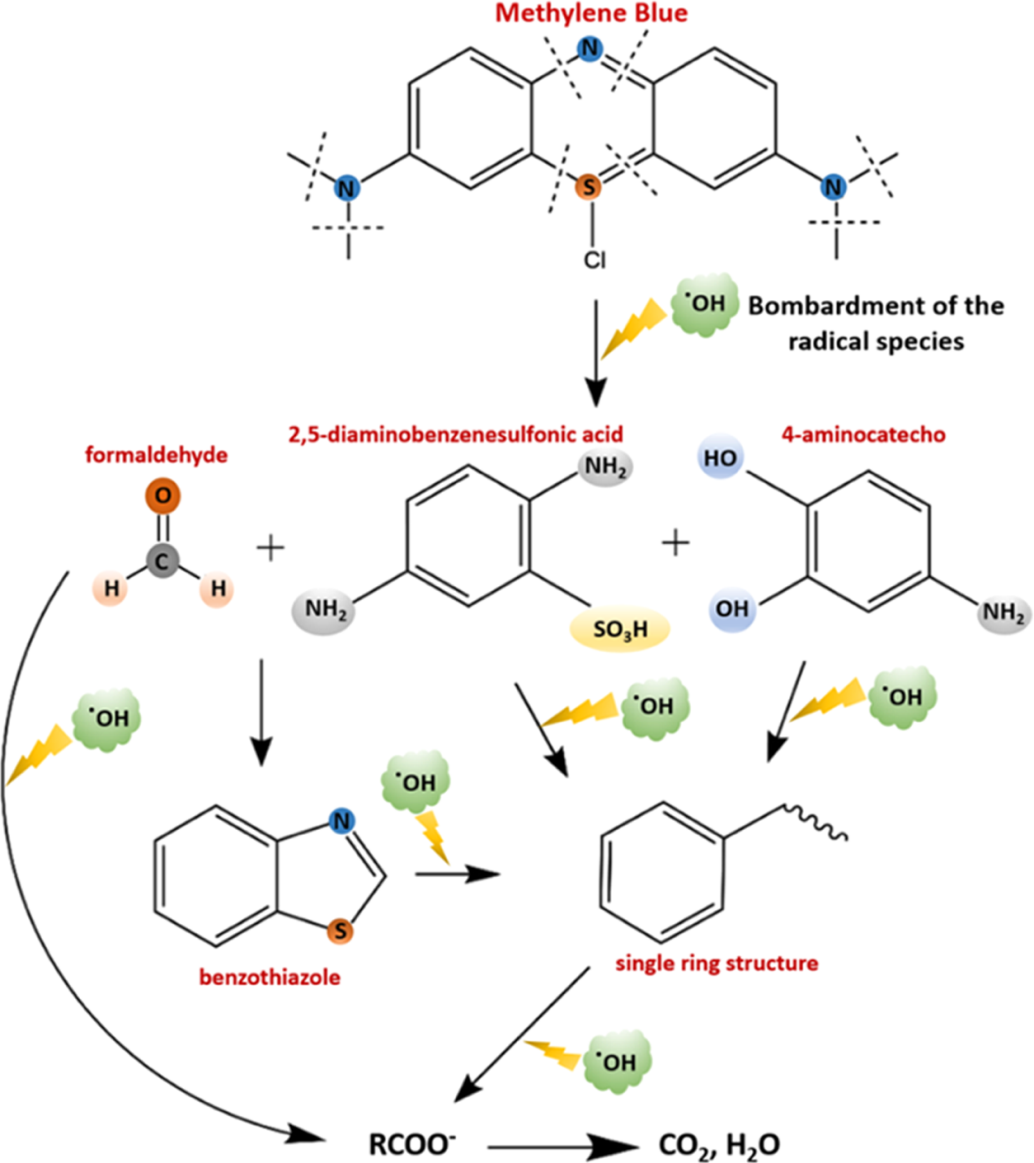
Possible MB degradation pathways using the EO process.

EO of pollutants with graphite electrodes allows two types of oxidation mechanisms (direct and indirect) with the generation of oxidants and free radicals in order to oxidize organic pollutants such as MB.^41^ In direct EO, formation of OH radicals by water on the anode surface occurs according to the given reaction
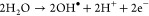
1When the supporting electrolytes such as sodium chloride were present in the solution, anodic oxidation facilitated conversion of chloride ions to chlorine and then to hypochlorite ions depending on the pH of water (eqs 2–4). Thus, strongly oxidant hypochlorite ions and highly reactive hydroxyl radicals formed as a result of the EO process facilitated reduction of the organic pollutants into intermediate products (Figure 1). Meanwhile, the only reaction that went on at the cathode side was conversion of water into hydroxyl ions and hydrogen (eq 5).
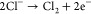
2
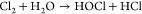
3
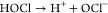
4
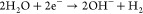
5According to the reaction pathways given above, MB removal in the EO system could be assumed to follow pseudo-first order kinetics (eq 6)^49^
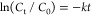
6where *C*_0_ and *C*_t_ are the initial and time = *t* concentrations of MB in mg/L, respectively, *k* is the rate constant in min^–1^, and t is time in min.

## 2 Materials and Methods

### 2.1 Materials

MB dye (C_16_H_18_N_3_SCl) was obtained from Fluka AG, reagent grade. The MB stock solution was prepared (1000 mg/L) and serial dilutions were made to obtain MB concentrations of 10, 30, and 50 mg/L. Solutions’ pH values were adjusted using 0.10 M NaOH or 0.10 M HCl. Reagent-grade sodium chloride (NaCl) was purchased from Fluka AG.

### 2.2 Experimental Method and Setup

**Figure 2:**
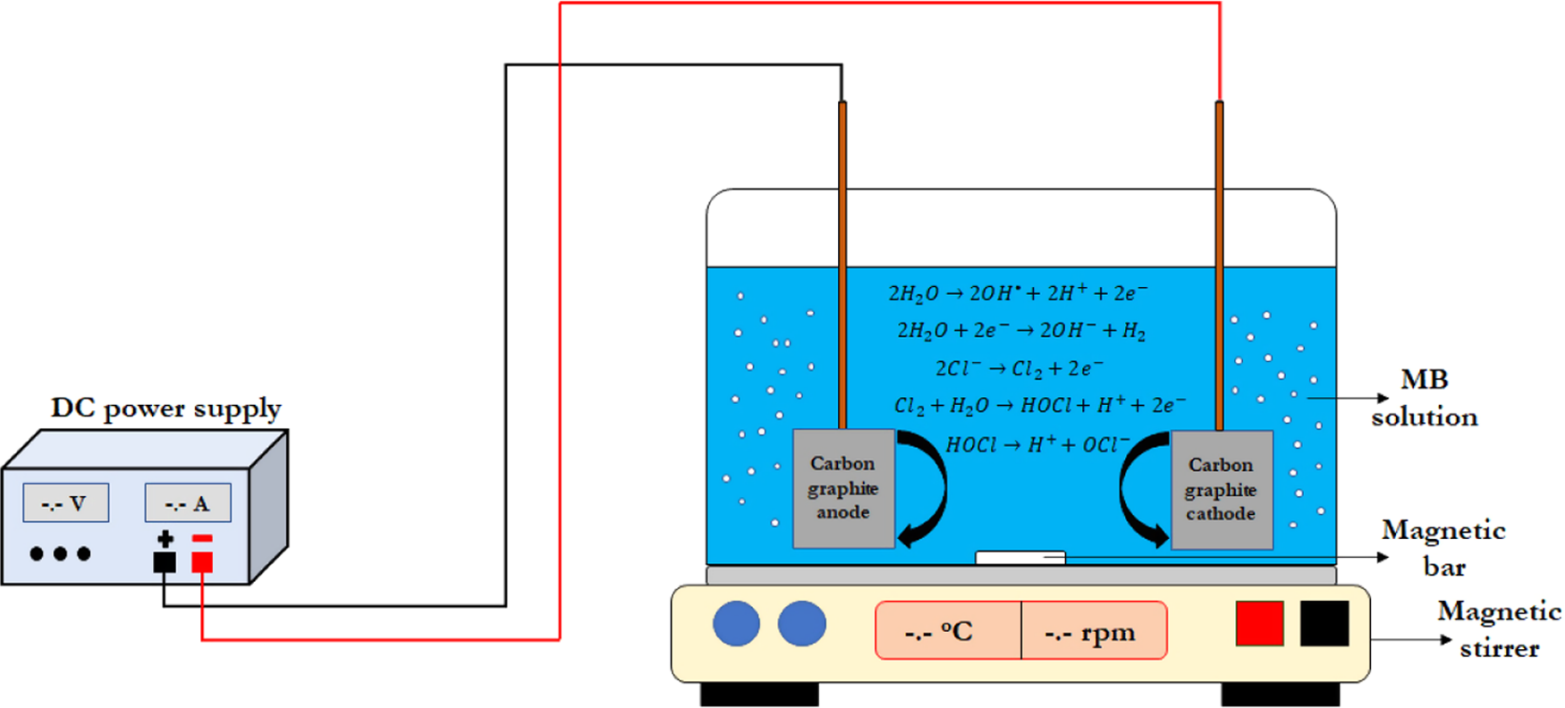
Experimental setup of the electrolysis cell.

Electrochemical degradation of MB was performed in a batch-type electrolysis cell. Flexible graphite electrodes (Sigma-Aldrich) were used as both anode and cathode, each with an area of 12 cm^2^ (6 cm × 2 cm), and electrodes were placed parallel to each other in the electrolysis cell. A flexible graphite electrode surface was observed using scanning electron microscopy (SEM, Quanta 250FEG) and atomic force microscopy (AFM) (Figure S1). It was also investigated for carbon content using energy dispersive X-ray spectrometry (EDX), which confirmed graphite material with a carbon content of 100%. The cathode to anode inter-electrode distance was 3 cm. The experimental setup of the electrolysis cell is shown in Figure 2.

The aqueous solution (50 mL) containing the MB dye was placed in the electrolysis cell. The flexible graphite anode and cathode were connected to a digital direct current (DC) power supply (Sunline, SL-3010D; 30V and 10A) and the desired electrical potential was supplied during the experiments. The electrolysis cell was equipped with a magnetic bar in order to provide thorough mixing. The temperature of the solution was kept constant at 25 °C during the experiments using a temperature-controlled magnetic stirrer plate. The EP (3–7 V), pH (4–10), *C*_*i*,MB_ (10–50 mg/L), *C*_*i*,NaCl_ (0.2–0.6 g/L), and *t* (30–90 min) ranges were specified in accordance with the literature.^50,51^ At the end of the operation, samples were taken from the cell and then analyzed spectrophotometrically.

### 2.3 Analytical Methods

A Benchtop pH analyzer (Mettler Toledo, SevenCompactTM) was used to measure the pH of the samples before and after the experimental procedures. MB concentrations were measured by a UV-spectrophotometer (Shimadzu, UV-2600) at a λ_max_ of 664 nm. All analyses were done in three replicates and the values were averaged. Standard deviation values were between 0.01 and 0.03 mg/L. eq 7 was used to determine the removal efficiency of MB
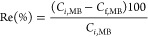
7where *C*_*i*,MB_ and *C*_f,MB_ are the initial and final MB dye concentrations (mg/L), respectively.

### 2.4 Box-Behnken Design and Data Analysis

The response surface methodology (RSM) with the BBD method was used as the experimental design tool. The BBD consisted of three main steps—determining the adequacy of the model, predicting the response variables, and assessing the minimum number of well-chosen statistically designed experimental runs (abbreviated as R). BBD was performed for the optimization of MB treatment to achieve high Re and low OC, and to study the individual and combined impacts of independent operational parameters on MB treatment. Design Expert 11 was performed for the statistical and data analysis. Five important operating variables (EP, pH*_i_*, *C*_*i*,MB_, *C_i_*,_NaCl_, and *t*_E_) were chosen as the independent operational parameters and were designated as *x*_1_, *x*_2_, *x*_3_, *x*_4_, and *x*_5_, respectively. The levels and ranges of the selected independent parameters are presented in Table 1. The independent operational parameters were considered at three levels coded as (−1), (0), and (+1) for low, center, and high, respectively.

**Table 1:** Independent Operational Parameters and Levels Conducted in the BBD

	range and levels		
independent variables	low (−1)	center (0)	high (+1)
*x*_1_: electrical potential (EP, V)	3.0	5.0	7.0
*x*_2_: initial pH (pH*_i_*)	4.0	7.0	10.0
*x*_3_: initial MB concentration (*C*_*i*,MB,_ mg/L)	10.0	30.0	50.0
*x*_4_: electrolyte dose (*C*_*i*,NaCl_, g/L)	0.2	0.4	0.6
*x*_5_: operating time (*t*, min)	30	60	90

The BBD procedure recommended forty-six experiments and arranged them with various combinations of independent operational parameters (Table S1). The MB dye Re (%), effluent MB dye concentration (*C*_f,MB_, mg/L), final pH of the solution (pH_f_), ENC (kWh/m^3^), and OC ($/m^3^) of the electrolysis cell were considered as the dependent operating variables (responses). The results of the dependent operating variables are presented in the Table S2. ENC was calculated using eq 8
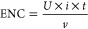
8where ENC is in kWh/m^3^, *U* is the cell electrical potential (V), *i* is the current (A), *v* is the reactor volume (m^3^), and *t* is the operating time (hour).

In the electrochemical treatment technology, the main factor contributing to OC was ENC. Therefore, the OC was calculated by considering the energy price using eq 9
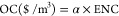
9where α represents the unit energy price (0.107 $/kWh). In this equation, market research was carried out, based on the price indexes dated March 2021.

## 3 Results and Discussion

### 3.1 Response Surface Methodology of Box-Behnken Modeling

In this study, BBD was conducted to obtain the effect of five independent operational parameters on the Re (% of MB dye), *C*_f,MB_ (mg/L), ENC (kWh/m^3^), and OC ($/m^3^). The results of the experiments for each operating variable are presented in the following sections. The accuracy of the model was statistically evaluated based on the test values presented in Table 2.

**Table 2:** Analysis of Variance of the Quadratic Model^a^

source	sum of squares	d*f*	mean squares	*F*-value	*p*-value	remarks
model	8779.19	20	438.96	6.31	<0.0001	significant
x_1_: electrical potential	3412.02	1	3412.02	49.03	<0.0001	significant
x_2_: initial pH	20.63	1	20.63	0.30	0.0909	insignificant
x_3_: initial MB concentration	3.58	1	3.58	0.05	0.0024	significant
x_4_: electrolyte dose	1290.25	1	1290.25	18.54	0.0002	significant
x_5_: operating time	613.18	1	613.18	8.81	0.0065	significant
residual	1739.88	25	69.60			
lack of fit	1738.28	20	86.91	271.72	<0.0001	significant
pure error	1.60	5	0.32			

a Fit statistics: Std. Dev = 8.34; mean = 90.61; CV% = 9.21; *R*^2^ = 0.8346; *R*_adjusted_^2^ = 0.7023; Adeq. Precision = 9.69.

Analysis of variance (ANOVA) was used to determine the statistical significance of the quadratic mathematical model. The coefficient of determination (*R*^2^) was used to determine the degree of fit and was defined as the proportion of variance in the dependent variable that could be explained by independent variables or as the ratio of explained variation to total variation.^52^*R*^2^ values greater than 0.7 indicated a satisfactory model fit, implying that the response model in this study could adequately explain the dye removal, with an *R*^2^ value of 0.84%. Additionally, statistical significance was attributed when the *p*-value and *F*-value were below 0.0001 and 123.7, respectively.^53^ Confirming statistical significance, the *p*-value and *F*-value of the studied model were found to be <0.0001 and 6.31, respectively. The coefficient of variation (CV) value of the model (9.21%) indicated that the model was reproducible as the CV value was below 10%. Consequently, the model results showed that the association between independent and dependent operating variables was well established with the mathematical model. The probability plots of residuals and externally studentized residuals for MB removal efficiency are provided in Figure S2.

Quadratic mathematical models were used to calculate the optimum parameters of the MB removal efficiency, *C*_f_,_MB_, ENC, and OC considering independent operational parameters (coded as factors *x*_1_–*x*_5_). The mathematical relationships between the independent operational parameters and responses are presented in eqs 10–13.
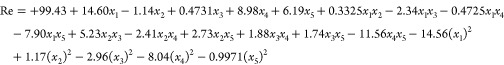
10
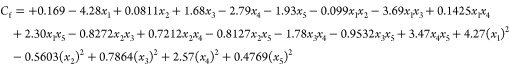
11
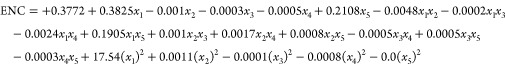
12
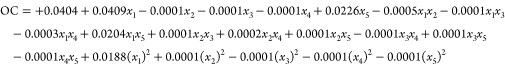
13

### 3.2 Effect of Electrical Potential

**Figure 3:**
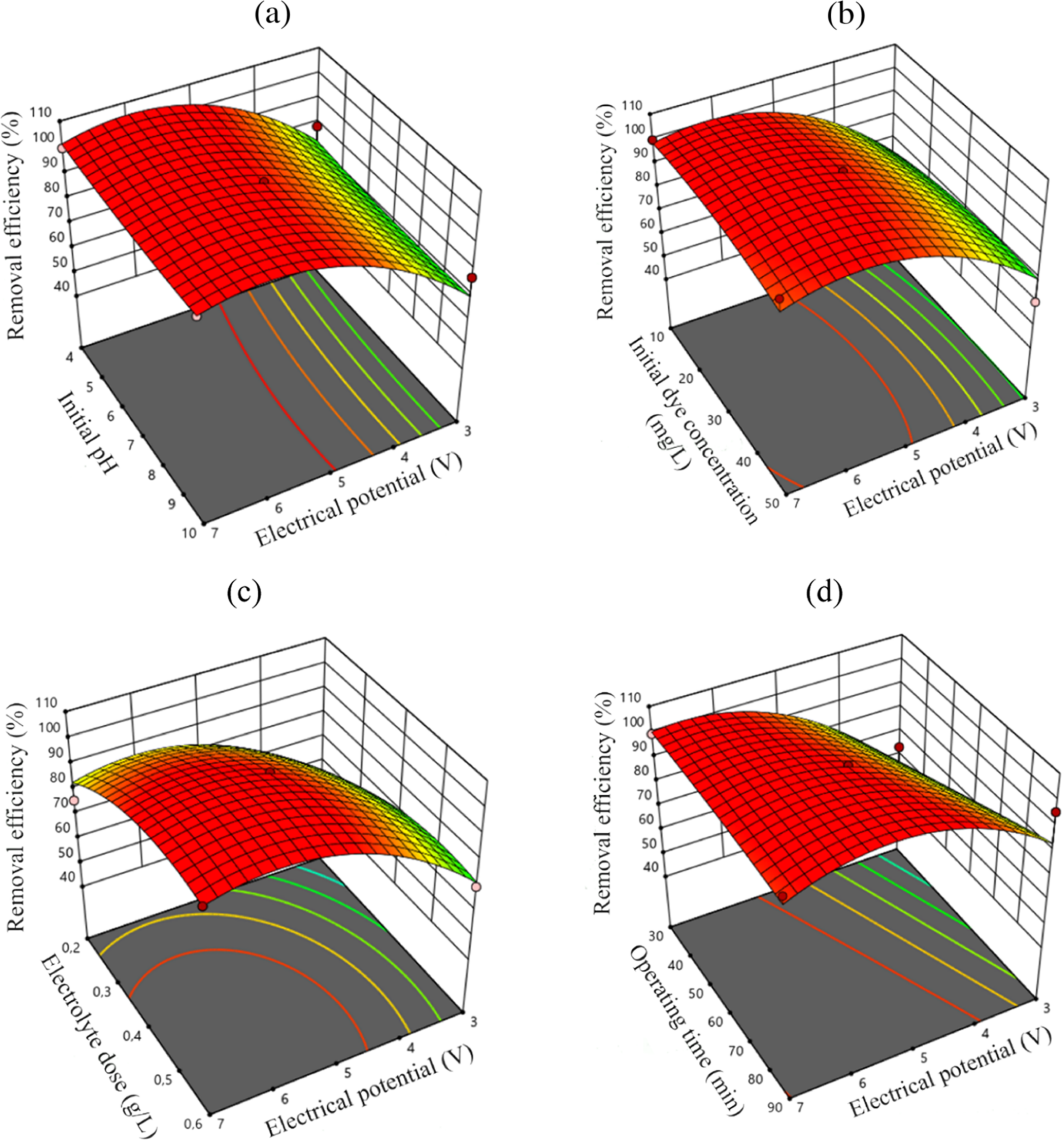
3D response surface graphs for the MB removal efficiency: (a) pH-EP, (b) *C*_*i*,MB_-EP, (c) *C*_*i*,NaCl_-EP, and (d) *t*-EP.

Being one of the most important parameters, electrical potential had a direct effect on the OC of the electrochemical process. Furthermore, the electrochemical reaction rates and thus the removal efficiency of electrochemical systems mainly depend on the electrical potential.^54^ High electrical voltage values must be avoided in electrochemical treatment processes considering the OC. Apart from the OC of the process, high electrical voltage values might cause the formation of undesirable byproducts, reactions, and attrition of electrode surfaces. In this study, in order to investigate the effect of EP on MB dye removal efficiency, experiments were performed with 3, 5, and 7 V, which corresponded to 0.25–0.58 A/cm^2^ in current density range. Figure 3 illustrates the combined influence of EP and other operational parameters on the effectiveness of MB removal. The three-dimensional (3D) contour plot depicting the combined effects of EP-pH (Figure 3a) and EP-*C*_*i*,MB_ (Figure 3b) showed that the MB removal efficiency was primarily affected by EP rather than pH or *C*_*i*,MB_. As the applied potential increased, the MB removal efficiency increased regardless of the pH value or *C*_*i*,MB_. On the other hand, the combined effect of *t*-EP increased the removal efficiency. The most pronounced combined effect was observed on the *C*_*i*,NaCl_-EP 3D contour plot. The MB removal efficiency increased from 48.8%, obtained with 3 V EP and 0.2 g/L *C*_*i*,NaCl_, to 100%, obtained with 7 V EP and 0.6 g/L *C*_*i*,NaCl_.

According to *p*-value and *F*-value results (*p*-value < 0.0001, *F*-value = 49.0), EP was the most significant factor for the MB dye removal. As expected, the effectiveness of removing MB improved as the EP increased owing to an increase in the formation of oxidants.^55,56^ At an initial MB concentration of 30 mg/L, the effectiveness of MB removal improved from 85.0 to 100% when the EP was increased from 3 to 6 V (Figure 3b). Similarly, MB removal efficiencies were found to be almost 80.0% (3 V) and 100% (>4 V) at a pH of 4 (Figure 3a). MB removal efficiencies were 99.4% (*C*_f,MB_: 0.18 mg/L) and 63.5% (*C*_f,MB_: 10.97 mg/L) at EP values of 7 and 3 V (R11 and R37) in Table S2, respectively, when other operating parameters were kept constant (pH: 7, *C*_*i*,MB_: 30 mg/L, *C*_*i*,NaCl_: 0.4 g/L, and *t*: 30 min). For the R11 and R37, the pH levels of treated water were 7.86 and 7.80, respectively. Similar results were observed for R15 and R23; the MB removal efficiency increased from 51.2% (*C*_f,MB_: 4.88 mg/L) to 99.8% (*C*_f,MB_: 0.02 mg/L) with the increasing EP (from 3 to 7 V). These results pointed to increased oxidant production (chlorine, hypochlorite) during the treatment process, and hence improved MB removal efficiency. The ENC and OC of the treatment process also increased with EP. The ENC and OC were 0.466 kWh/m^3^ and 0.0498 $/m^3^ at an EP of 7 V, and 0.084 kWh/m^3^ and 0.009 $/m^3^ at an EP of 3 V.

### 3.3 Effect of Initial pH

**Figure 4:**
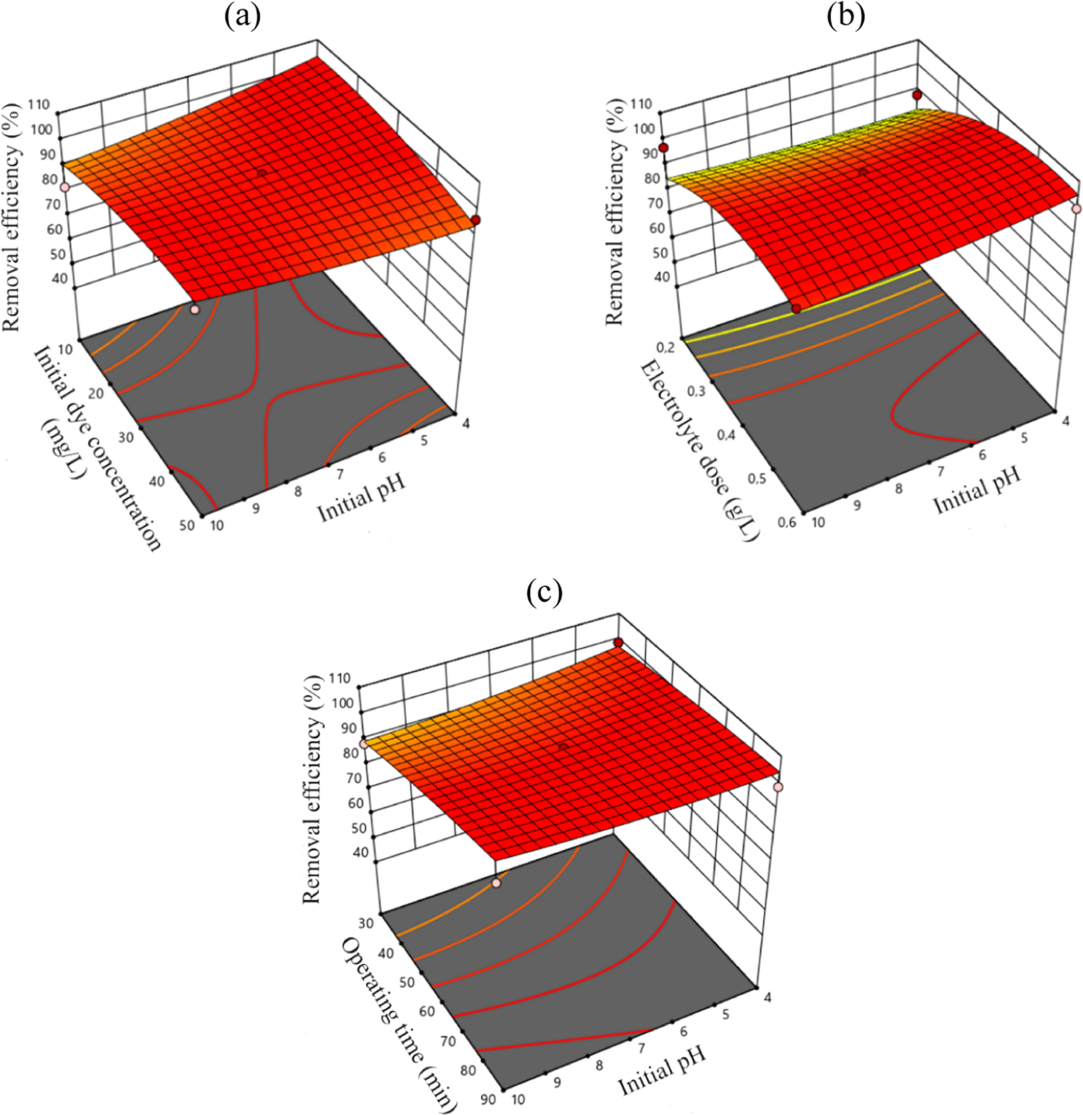
3D response surface graphs for MB removal efficiency: (a) pH-*C*_*i*,MB_, (b) pH-*C*_*i*,NaCl_, and (c) pH-*t*.

The effect of pH (4, 7, and 10) of the aqueous solution on dye removal was investigated (Table S1) since it affected the performance of the electrochemical treatment processes depending on the electrolyte type, investigated organic pollutant, and electrode material. For different *C*_*i*,MB_ values, the response of the MB removal efficiency varied as the initial solution pH increased (Figure 4a). While the removal effectiveness dropped from 100 to 92% when the pH was increased from 4 to 10, it increased as the pH increased for higher MB dye concentrations (30–50 mg/L). However, it should be noted that the increase was minimal. At a *C*_*i*,MB_ of 50 mg/L, the MB removal efficiency was 96.4% at a pH of 4, while it was 99.4% at a pH of 10 (R14 and R1 in Table S2, respectively). When the pH value was kept constant, the removal performance deteriorated slightly as the initial dye concentration increased within the acidic pH range. In the alkaline pH range, the removal efficiency did not change for varying initial dye concentrations. MB removal efficiencies were found to be 77.3% (*C*_f,MB_: 6.80 mg/L) and 78.8% (*C*_f,MB_: 6.35 mg/L) at initial pH values of 10 and 4 (R6 and R20), as shown in Table S2, respectively, when the other operating parameters were the same. These results demonstrated that the MB dye removal efficiency did not correlate significantly within the studied pH range.

The combined effect of *C*_*i*,NaCl_ and pH showed that increasing the electrolyte dose improved the removal performance at all of the studied pH values (Figure 4b), with high electrolyte doses (>0.4 g/L) yielding a dye removal efficiency of higher than 90.0%. Expectedly, the MB dye removal efficiency increased as the operating time increased at all pH values (Figure 4c); however, the increase was significant for alkaline pH values. 99.5% (R8) and 88.6% (R4) removal efficiencies were calculated for 90 min and 30 min operation times, respectively (Tables S1 and S2). At longer operating times (i.e., 90 min), different pH values yielded very similar and more than 98.0% removal efficiencies (R8 and R38).

Three possible chloride species (Cl_2_, ClOH, and ClO^–^) well known as “active chlorine” constituted the global concentration of the dissolved chlorine in solution after the chlorination process. Cl_2_ was previously known to be the major species in the acidic zone; the ClOH species predominate in the 3.3–7.5 pH range, and ClO^–^ ions should be the dominant species above pH 7.5. As a result, the anode might produce Cl_2_ and ClOH species in acidic solution, resulting in high rates of MB removal.^19^ Furthermore, at alkaline pH values, the conductivity of the aqueous solution decreased due to increasing electrolyte consumption, and thus, the removal efficiency of the organic contaminants decreased. For instance, Samarghandi et al. investigated the effects of operating parameters on MB dye removal using an electrochemical degradation process with graphite-doped PbO_2_ anode.^57^ They reported the maximum MB removal efficiency at an operating time of 50 min to be 97.7% at a pH of 5.75, while the minimum removal efficiency was 79.7% at an initial pH of 11. In a separate study, Baddouh et al. investigated the MB removal from aqueous solutions by electrochemical treatment with Ti/RuO_2_–IrO_2_ and SnO_2_ electrodes.^51^ They observed 100% MB removal efficiency at acidic conditions (pH 3), *j* = 40 mA/cm^2^, [NaCl] = 0.1 mol/L, [MB] = 100 mg/L, and *T* = 25 °C using the Ti/RuO_2_–IrO_2_ electrode. Although acidic conditions performed better at lower initial dye concentrations when the other operating parameters were kept constant, our results did not point to a clear acidic range preference in terms of removal efficiency.

The 3D contour plots in Figures 3a and 4 show the combined effects of independent operating parameters on the MB removal efficiency at different pH values. These results suggested that electrical potential had a greater influence on the MB removal efficiency than the solution’s initial pH indicated. The combined impact of pH-*t* and *C*_*i*,NaCl_-pH of the solution exhibits a similar pattern. Moreover, the ENC and OC of the process were not significantly affected by the pH of the solution. For instance, the OC values were found to be 0.0182 and 0.0184 $/m^3^ at pH values of 10 and 4, respectively (R6 and R20). Our results showed that the electrochemical treatment of the MB dye using the carbon electrode was promising in terms of both dye removal efficiency and ENC compared with the literature. Baddouh et al. reported that the specific ENC of the electrochemical MB treatment process using the Ti/RuO_2_–IrO_2_ electrode was in the range of 1.320 and 3.348 kWh/m^3^ under different operating conditions.^51^

### 3.4 Effect of the Initial Dye Concentration

The removal efficiency increased when the initial dye concentration increased while all other operational parameters remained unchanged (Tables S1 and S2—R1 and R5). The MB removal efficiency was 81.9% (*C*_f,MB_: 1.81 mg/L) at a concentration of 10 mg/L, while it was 99.4% (*C*_f,MB_: 0.30 mg/L) at a concentration of 50 mg/L. The highest removal (∼95%) was achieved at 0.40 g/L electrolyte dose. Concentration acted as a major driving force in overcoming the mass transfer resistance, with a higher starting dye concentration implying an accelerated degradation rate.^58^

**Figure 5:**
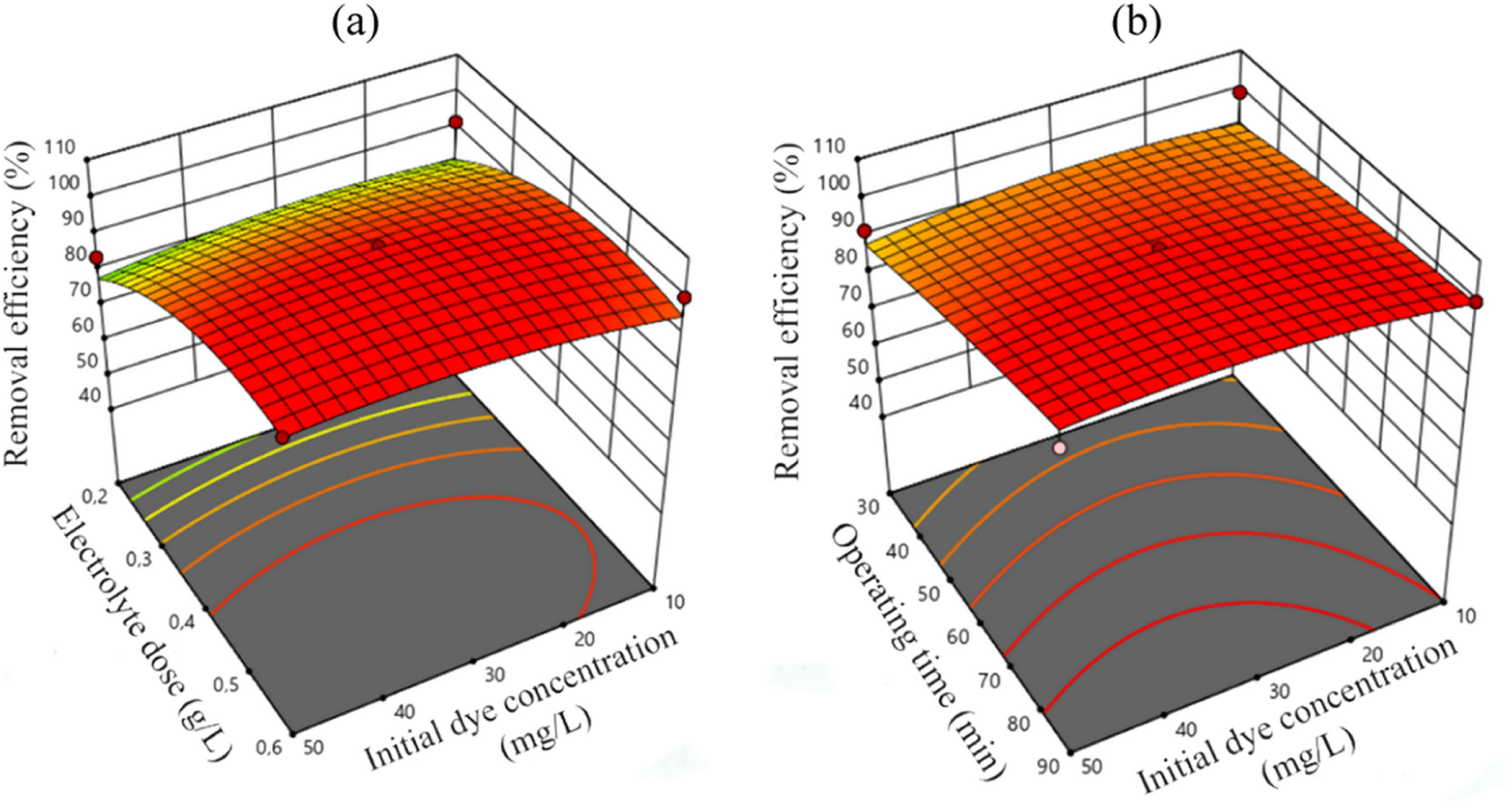
3D response surface graphs for MB removal efficiency: (a) electrolyte dose–initial MB concentration, (b) initial MB concentration–operating time.

The combined effect of electrolyte dose–initial dye concentration on the removal of MB is demonstrated in Figure 5a. As the electrolyte dose was increased, the removal efficiency increased for all initial dye concentrations. For a t of 60 min, EP of 5 V, initial pH of 10, and *C*_*i*,MB_ of 30 mg/L, increasing the *C*_*i*,NaCl_ from 0.2 to 0.6 g/L yielded an increase in removal efficiency from 96.97% (R25) to 99.13% (R3). While keeping *t* and EP the same, the same increase in electrolyte dose for a pH of 7 and *C*_*i*,MB_ of 50 mg/L (R7 and R10) yielded a much more significant increase (from 84 to 99.95%). Expectedly, increasing *t* increased the removal efficiency for all *C*_*i*,MB_ (Figure 5b). As the operating time progressed, the removal efficiency increased at all initial dye concentrations due to continuous energy consumption to produce electrons necessary for MB oxidation.

The ENC and OC values at different initial dye concentrations revealed that the initial dye concentration did not significantly affect these parameters. For instance, the ENC and OC were found to be 0.17 kWh/m^3^ and 0.061 $/m^3^ at both *C*_*i*,MB_ values of 10 and 50 mg/L under the same operating conditions (runs 21 and 23). Similar results were observed for experimental runs of R15 and R44, R2 and R7, and R14 and 16 (Table S2).

### 3.5 Effect of Electrolyte Dose

**Figure 6:**
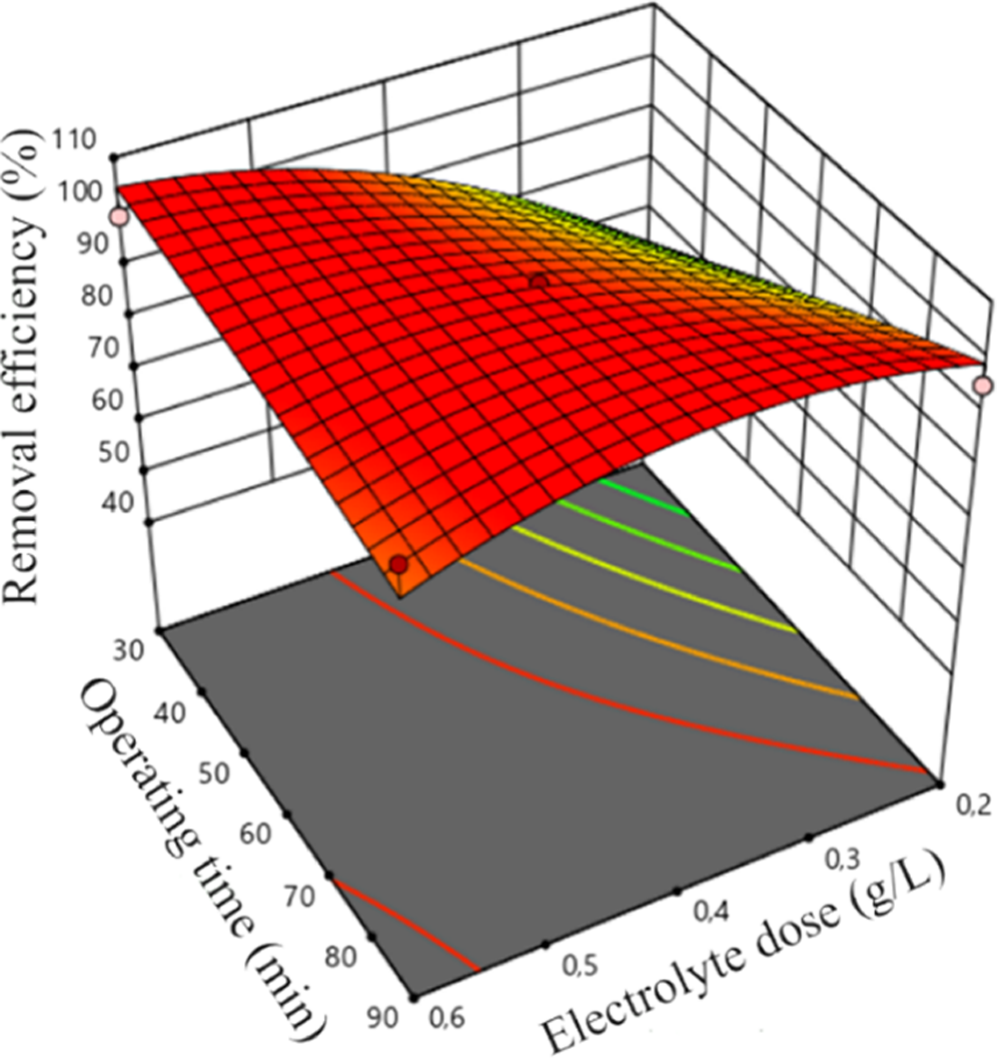
3D response surface graphs for MB removal efficiency: *t*-*C*_*i*,NaCl_.

The electrolytes in electrochemical treatment processes improved the ionic strength of the aqueous solution by increasing the electrical current transfer in solution. NaCl, Na_2_SO_4_, KCl, and NH_4_Cl were widely used in electrochemical processes.^41^ As expected, the MB removal efficiency improved as the NaCl content increased. The removal efficiency increased from 48.7% (*C*_f,MB_: 15.39 mg/L) to 99.5% (*C*_f,MB_: 0.16 mg/L) when the electrolyte concentration was tripled from 0.20 g/L at a pH of 7, EP of 5 V, *C*_i,MB_ of 30 mg/L, and *t* of 60 min (R32 and R33) (Table S2). Similar trends were observed for some other experimental runs (R43 and R46, R40 and R41, Table S2). The 3D surface plots that have been presented so far in Figures 3c, 4b, and 5a show the MB removal efficiencies for different *C*_*i*,NaCl_ values. For instance, the MB removal efficiencies were 80.0 and 100% at *C*_*i*,NaCl_ values of 0.2 and 0.6 g/L under constant operating conditions (pH: 4, EP: 5 V, *C*_*i*,MB_: 30 mg/L, and *t*: 60 min), respectively (Figure 3c). The removal efficiency of MB increased from 85.0 to 100% at *C*_*i*,NaCl_ values of 0.2 and 0.6 g/L under constant operating conditions (pH: 4, EP: 3 V, *C*_*i*,MB_: 30 mg/L, and *t*: 60 min), respectively (Figure 4b). A similar trend was observed for the different operating conditions shown in Figures 5a and 6. This was most probably due to two important phenomena occurring simultaneously at the anode surface: (1) increased production of strong oxidation species such as Cl_2_, OCl^–^, and ClOH and (2) increase in electrical conductivity of the solution on increasing the production of OH radicals.^57^

Under the combined effect of *C*_*i*,NaCl_ and EP, the MB removal efficiency was 77.3% (*C*_f,MB_: 6.80 mg/L) at 3 V of EP and 0.4 g/L of *C*_*i*,NaCl_ and it was 99.1% (*C*_f,MB_: 0.26 mg/L) at 5 V of EP and 0.6 g/L of *C*_*i*,NaCl_ (R3 and R6). The results showed that simultaneous increase in EP and *C*_*i*,NaCl_ increased the MB dye removal efficiency. As expected, the ENC and OC of the process increased with the simultaneous increase in *C*_*i*,NaCl_ and EP. ENC values were calculated as 0.170 and 0.379 kWh/m^3^, while the OCs were 0.0182 and 0.0406 $/m^3^ in R6 and R3, respectively.

### 3.6 Effect of Operational Time

In electrochemical treatment processes, operational time was another important parameter that directly affected removal efficiency. The ENC, oxidation agent formation, and OC mainly depended on the operational time. Therefore, the effect of t on the MB removal efficiencies under different operating conditions was investigated in this study (Figures 3–6). The MB removal efficiency significantly increased with rising t. At the same operating conditions, the removal efficiencies were 63.5% (*C*_f,MB_: 10.95 mg/L) and 95.5% (*C*_f,MB_: 1.64 mg/L) for *t* of 30 and 90 min, respectively (R13 and R37). The reason for enhancement of the MB removal efficiency at high operating temperatures was the prevention of oxidation agents’ production in solution. Increased *t*, on the other hand, had a drastic effect on the ENC and OC values of the process. The ENC and OC increased from 0.084 to 0.253 kWh/m^3^ and from 0.009 to 0.0271 $/m^3^ at *t* of 30 and 90 min, respectively (R13 and R37).

Furthermore, the reaction kinetics revealed some interesting results (Table 3). It was apparent that an increase in the *C*_*i*,MB_ from 10 to 30 mg/L resulted in an increase in the reaction kinetics, but further increase in the concentration from 30 to 50 mg/L led to a decrease in the reaction kinetics. At lower MB concentrations, the rate of EO was higher than the rate of MB mass transfer to the anode surface, allowing all MB to be oxidized. However, further increase in the initial MB concentration caused more MB to get closer to the anode surface, and this time the oxidation agent concentration became the rate-limiting factor, and thus, the reaction kinetics slowed down. Further, more intermediates competing with the parent molecules of MB might get adsorbed onto the anode surface, which might lead to inactivation of the electrocatalyst sites. The increase in EP was in good agreement with the increase in the reaction kinetics since simultaneous generation of strong oxidation species and OH radicals at the anode surface occurred, enhancing the MB degradation. The reaction kinetics was observed to be at its maximum for pH 5, and as the pH increased, the reaction rates decreased.

**Table 3:** Kinetic Rate Constants of the Electrochemical Removal of MB Dye in the EO System Using the Flexible Graphite Anode for Some Parameters

initial concentration (mg/L)	10	20	30	40	50
*k* (min^–1^)	0.0576	0.0734	0.0760	0.0689	0.0293

electrical potential (V)	3	4	5	6	7
*k* (min^–1^)	0.0208	0.0396	0.0852	0.1354	0.1454

pH	4	5	6	7	8
*k* (min^–1^)	0.0958	0.1015	0.0875	0.0877	0.0847

### 3.7 Operating Cost Evaluation

**Figure 7:**
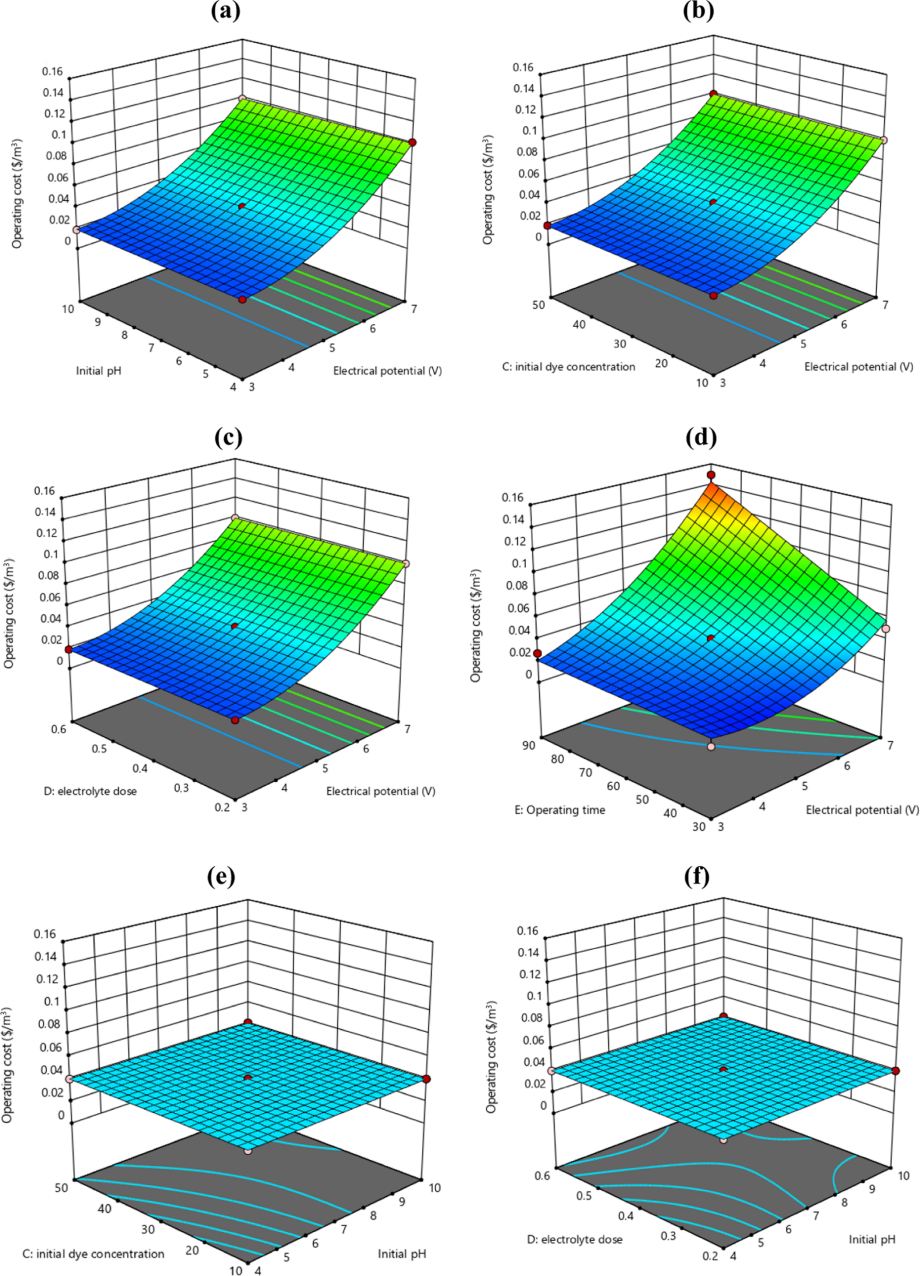
3D response surface graphs for the MB removal efficiency on the OC: (a) pH-EP, (b) *C*_*i*,MB_-EP, (c) *C*_*i*,NaCl_-EC, (d) *t*-EP, (e) *C*_*i*,MB_-pH, and (f) *C*_*i*,NaCl_-pH.

According to literature, energy consumption directly affected the OC of the electrolysis system.^59,60^ Therefore, the OC of each experimental run was calculated to investigate the techno-economic effects of the operating variables. The results showed that the OC of the system was not affected by *C*_*i*,NaCl_ or *C*_*i*,MB_. For instance, the OC of the system was 0.0608 and 0.0607 $/m^3^ for *C*_*i*,NaCl_ of 0.2 and 0.6 g/L, respectively (R40 and R41, Table S2). Similarly, an OC of 0.0201 $/m^3^ was observed for *C*_*i*,NaCl_ of 0.2 and 0.6 g/L in R32 and R33. For *C*_*i*,MB_ of 10 and 50 mg/L, the OC was 0.1001 $/m^3^ at an EP of 7 V, *t* of 60 min, pH of 7, and *C*_*i*,NaCl_ of 0.4 g/L. On the other hand, the OC was significantly affected by the pH and EP of the system. The OC increased with increasing EP due to high ENC. The OC of the system increased from 0.0271 to 0.1494 $/m^3^ on increasing the EP from 3 to 5 V (R13 and R28). Furthermore, the combined effects of the operating variables on the OC were also evaluated, and the 3D plots are presented in Figure 7. As expected, the OC of the system significantly decreased from 0.0401 to 0.009 $/m^3^ with the simultaneous decreases in EP and *t* from 5 to 3 V and 60 to 30 min (R36 and R37), respectively. On the other hand, there was no significant change in the OC of the system under the combined effects of pH-*C*_*i*,MB_ and pH-*C*_*i*,NaCl_ (Figure 7e,f).

### 3.8 Optimization of Operational Parameters

Optimization of the EO process was the most important goal of this study. In the optimization, the BBD was conducted to optimize the values of the dependent operating conditions within the specified values to achieve maximum MB removal efficiency and minimum OC. The optimum operating parameters were found to be as follows: pH: 4, *C*_*i*,MB_: 26.5 mg/L, *C*_*i,*NaCl_: 0.6 g/L, EP: 3 V, and *t*: 30 min. Responses of the *C*_f.MB_, Re, ENC, and OC were 0.001 mg/L, 99.9%, 0.178 kWh/m^3^, and 0.012 $/m^3^, respectively. A detailed comparison of the MB removal efficiency with the literature in terms of *t*, electrode type, and process efficiency is given in Table 4.

**Table 4:** Comparison of This Study with Literature^a^

method	anode	cathode	operating conditions	removal efficiency (%)	references
EC-ECa	graphite	SSM, Al/SSM, Ti/SSM	pH*_i_*:5.5, CD: 25 mA/cm^2^, *t*_o_: 20 min, *C*_*i*,MB_: 25 mg/L, *C*_*i*,NaCl_: 0.15 M	88.0	(47)
EO	iron	graphite	pH*_i_*: 3, V: 9 V, *t*_o_: 60 min, *C*_*i*,MB_: 10 mg/L, *C*_*i*,Na_2_SO_4__: 0.1 M	86.5	(34)
EO	graphite	graphite	pH*_i_*: 3, V: 5 V, *t*_o_: 5 h, *C*_*i*,MB_: 10 mg/L	99.2	(50)
EO	Pb/PbO_2_	stainless steel	pH*_i_*: 7.6, CD: 23 mA/cm^2^, *t*_o_: 60 min, *C*_*i*,MB_: 0.155 mg/L	94.7	(56)
AO	SnO_2_		pH*_i_*: 3, CD: 60 mA/cm^2^, *t*_o_: 30 min, *C*_*i*,MB_: 100 mg/L	≈100	(41)
EO	Ti/RuO_2_–IrO_2_ and SnO_2_	platinum plaque	pH*_i_*: 3, CD: 40 mA/cm^2^, *t*_o_: 10 min, *C*_*i*,MB_: 100 mg/L, *C*_i,NaCl_: 0.1 M	≈100	(51)
ED	PbO_2_	stainless steel	pH*_i_*: 3, CD: 50–70 mA/cm^2^, *t*_o_: 60 min, *C*_*i*,MB_: 20–400 mg/L, *C*_*i*,Na2SO4_: 2–12.8 g/L	99.4	(61)
AO	Pb/PbO_2_	Pb or SS	pH*_i_*: 7, CD: 50 mA/cm^2^, *t*_o_: 180 min, *C*_*i*,MB_: 10 mg/L	89.5	(62)
EO	graphite	graphite	pH*_i_*: 4, V: 5 V, *t*_o_: 60 min, *C*_*i*,MB_: 30 mg/L, *C*_*i*,NaCl_: 0.6 g/L	≈100	in this study

a EC: electrocoagulation, ECa: electrocatalysis, EO: electrochemical oxidation, AO: anodic oxidation, ED: electrochemical degradation.

## 4 Conclusions

In this study, forty-six experimental runs were conducted using the BBD approach in the electrochemical treatment process for investigation of various operating parameters’ effects on MB dye removal. Significantly positive effects of EP and *C*_*i*,NaCl_ on the MB removal efficiency were observed. Likewise, MB removal efficiency increased with increasing *t* and increasing *C*_*i*,MB_. On the other hand, the opposite observations were made regarding the effect of pH on MB removal efficiency. While removal efficiency decreased with increasing pH values for low dye concentrations (R5 and R16), it increased with increasing pH values for high dye concentrations (R25 and R46). *t*-EP and EP-*C*_*i*,NaCl_ combined had a positive effect on the MB removal efficiency, while the interactions of other operating variables combined did not have considerable effects on the removal efficiency. Furthermore, EP was found as the most effective parameter for the OC of the system. Consequently, the highest Re and lowest OC were found to be 99.9% and 0.012 $/m^3^ for a *t* of 30 min, pH of 4, *C*_*i*,MB_ of 26.5 mg/L, *C*_*i*,NaCl_ of 0.6 g/L, and EP of 3 V. The results revealed that electrochemical treatment using flexible graphite was a promising treatment technology for effective MB removal from aqueous solution with high removal performance, low *t*, and minimum OC.

## Supporting Information Available

Box-Behnken design results, SEM, and AFM images of graphite electrode (PDF)
