# Supplementary material for: Electrochemical Degradation of Methylene Blue by a Flexible Graphite Electrode: Techno-Economic Evaluation
Source: ACS Omega. 2022 Sep 2;7(36):32640–52. doi: 10.1021/acsomega.2c04304 (PMC9476165; doi:10.1021/acsomega.2c04304)
Supplement: Supplementary file 1 — ao2c04304_si_001.pdf [file ao2c04304_si_001.pdf]

Supporting Information

Electrochemical degradation of methylene blue by flexible graphite electrode: Techno-economic evaluation

Aysegul Yagmur GOREN[1], Yaşar Kemal RECEPOĞLU[2], Özge EDEBALİ[1], Cagri SAHIN[1], Mesut GENISOGLU[1], Hatice Eser OKTEN[1,3*]

[1] Izmir Institute of Technology, Department of Environmental Engineering, İzmir 35430, Turkey

[2] Izmir Institute of Technology, Department of Chemical Engineering, İzmir 35430, Turkey

[3] İzmir Institute of Technology, Environmental Development Application and Research Centre, İzmir 35430, Turkey

*Corresponding Author: haticeokten@iyte.edu.tr

Table S1. BBD with independent operating variables.

Run no.	Electrical potential (V)	Initial pH (-)	Initial dye concentration (mg/L)	Electrolyte dose (g/L)	Operating time (min)
1	5	10	50	0.4	60
2	5	7	10	0.2	60
3	5	10	30	0.6	60
4	5	10	30	0.4	30
5	5	10	10	0.4	60
6	3	10	30	0.4	60
7	5	7	50	0.2	60
8	5	10	30	0.4	90
9	5	7	30	0.4	60
10	5	7	50	0.6	60
11	7	7	30	0.4	30
12	5	7	30	0.4	60
13	3	7	30	0.4	90
14	5	4	50	0.4	60
15	7	7	10	0.4	60
16	5	4	10	0.4	60
17	7	7	30	0.6	60
18	5	7	10	0.4	30
19	3	7	30	0.2	60
20	3	4	30	0.4	60
21	3	7	50	0.4	60
22	5	7	10	0.4	90
23	3	7	10	0.4	60
24	5	7	30	0.4	60
25	5	10	30	0.2	60
26	5	7	30	0.4	60
27	7	7	30	0.2	60
28	7	7	30	0.4	90
29	5	7	50	0.4	90
30	5	7	30	0.4	60
31	5	7	10	0.6	60
32	5	7	30	0.6	30
33	5	7	30	0.2	30
34	7	10	30	0.4	60
35	5	7	50	0.4	30
36	5	7	30	0.4	60
37	3	7	30	0.4	30
38	5	4	30	0.4	90
39	5	4	30	0.4	30
40	5	7	30	0.2	90
41	5	7	30	0.6	90
42	7	4	30	0.4	60
43	5	4	30	0.6	60
44	7	7	50	0.4	60
45	3	7	30	0.6	60
46	5	4	30	0.2	60

Table S2. BBD with responses.

Run no.	$C_{f,MB}$ (mg/L)	Final pH (-)	Removal efficiency (%)	ENC (kWh/m^3)	Operating cost ($/m^3)
1	0.301	10.89	99.40	0.379	0.0406
2	0.842	7.91	91.58	0.376	0.0402
3	0.261	10.56	99.13	0.379	0.0406
4	3.422	11.12	88.59	0.189	0.0202
5	1.808	10.93	81.92	0.377	0.0403
6	6.804	11.02	77.32	0.170	0.0182
7	8.001	7.85	83.99	0.375	0.0401
8	0.138	11.25	99.54	0.567	0.0607
9	0.359	7.59	98.80	0.377	0.0403
10	0.027	7.65	99.95	0.372	0.0398
11	0.181	7.86	99.40	0.466	0.0498
12	0.026	7.53	99.91	0.379	0.0406
13	1.637	7.89	95.54	0.253	0.0271
14	1.821	6.21	96.36	0.376	0.0402
15	0.021	7.92	99.79	0.935	0.1001
16	0.019	6.18	99.81	0.378	0.0404
17	0.007	7.73	99.98	0.931	0.0996
18	0.096	8.01	99.04	0.190	0.0203
19	16.81	8.12	43.97	0.168	0.0180
20	6.355	5.97	78.82	0.172	0.0184
21	19.84	8.03	60.31	0.172	0.0184
22	0.011	7.65	99.89	0.569	0.0608
23	4.883	7.72	51.17	0.171	0.0183
24	0.399	8.10	98.67	0.375	0.0401
25	0.910	10.52	96.97	0.376	0.0402
26	0.123	7.59	99.59	0.379	0.0406
27	7.227	7.21	75.91	0.937	0.1002
28	0.050	7.86	99.83	1.397	0.1494
29	0.258	7.58	99.48	0.569	0.0608
30	0	7.61	100	0.378	0.0404
31	0	7.72	100	0.375	0.0401
32	0.155	7.92	99.48	0.188	0.0201
33	15.393	7.86	48.69	0.189	0.0202
34	0.070	10.38	99.77	0.930	0.0995
35	4.156	7.30	91.69	0.188	0.0201
36	0.107	7.56	99.64	0.375	0.0401
37	10.95	7.80	63.5	0.084	0.0090
38	0.319	6.22	98.84	0.564	0.0603
39	0.352	6.38	98.82	0.189	0.0202
40	1.411	7.65	95.30	0.569	0.0608
41	0.048	7.71	99.84	0.567	0.0607
42	0.017	6.50	99.94	0.951	0.1017
43	0	6.70	100	0.375	0.0401
44	0.203	7.78	99.59	0.935	0.1001
45	9.020	7.65	69.93	0.172	0.0184
46	3.534	6.02	88.22	0.379	0.0406

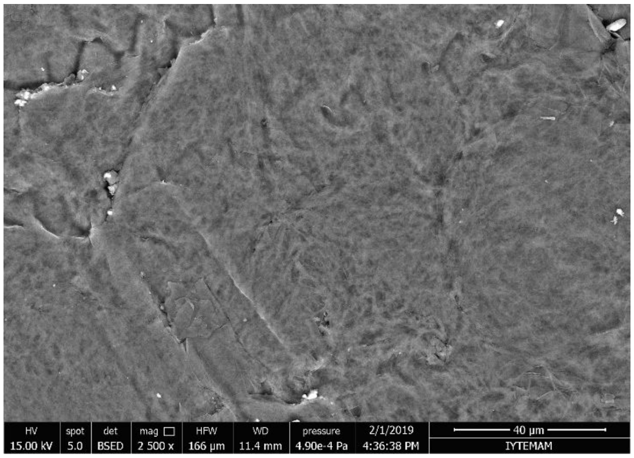

(a)

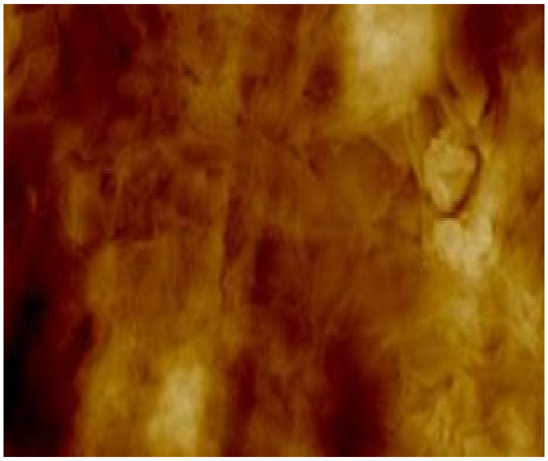

(b)

Figure S1. SEM (a) and AFM images (b) of commercially purchased graphite electrode.

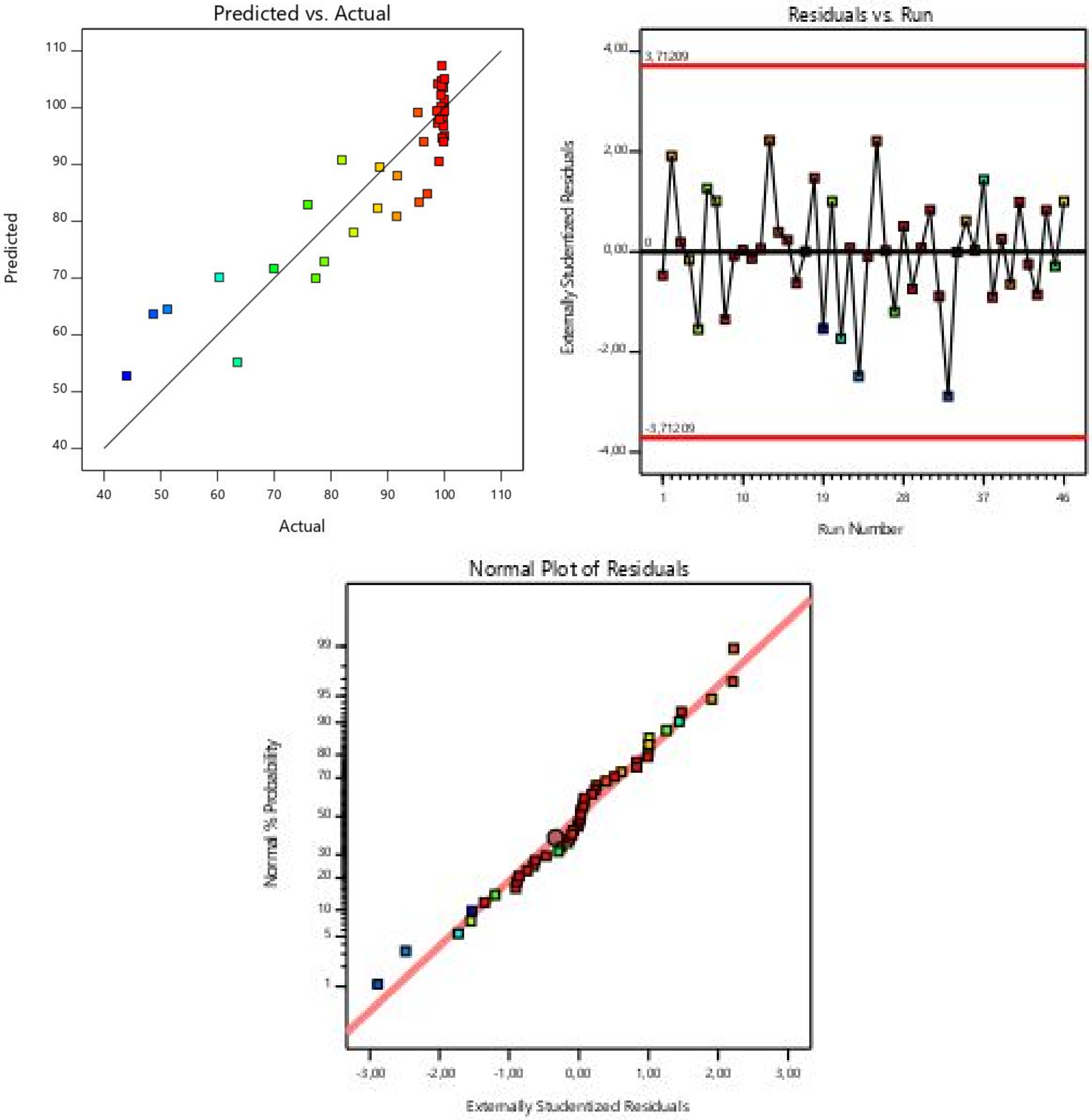

Figure S2. Statistical plots for MB dye removal: (a) Actual values against predicted ones, (b) The externally studentized residuals against run number, and (c) Normal plot of residuals.
